# Chitinolytic assay of indigenous *Trichoderma* isolates collected from different geographical locations of Chhattisgarh in Central India

**DOI:** 10.1186/2193-1801-1-73

**Published:** 2012-12-20

**Authors:** Toshy Agrawal, Anil S Kotasthane

**Affiliations:** Department of Plant Molecular Biology and Biotechnology, Indira Gandhi Krishi Vishwavidyalaya, Raipur, 492 006 Chhattisgarh, India

**Keywords:** Bromo cresol purple, Chitin, N-acetyl-β-D-glucosamine, p-nitrophenol, *Trichoderma*, Volume activity

## Abstract

Chitin is the second most abundant polymer in nature after cellulose and plays a major role in fungal cell walls. As a producer of variety of chitinase enzymes *Trichoderma* has become an important means of biological control of fungal diseases. A simple and sensitive method based on the use of basal medium with colloidal chitin as sole carbon source supplemented with Bromo cresol purple (pH indicator dye) is proposed to evaluate large populations of *Trichoderma* for chitinase activity. The soluble substrate with pH indicator dye (Bromo cresol purple, BCP) for the assay of chitinase activity on solid media is sensitive, easy, reproducible semi-quantitative enzyme diffusion plate assay and economic option to determine chitinases. Colloidal chitin derived from *Rhizoctonia* cell wall and commercial chitin included as a carbon source in broth also allowed selection and comparison of chitinolytic and exochitinase activity in *Trichoderma* spectrophotometrically. Released N-acetyl-β--D-glucosamine (NAGA) ranged from 37.67 to 174.33 mg/ml and 37.67 to 327.67 mg/ml and p-nitrophenol (pNP) ranged from 0.17 to 35.78 X 10^-3^ U/ml and 0.62 to 32.6 X 10^-3^ U/ml) respectively with *Rhizoctonia* cell wall and commercial chitin derived colloidal chitin supplemented broth.

## Background

Efficient bio-control strains of the genus *Trichoderma* are being developed as promising biological fungicides and their weaponry for this function also includes secondary metabolites with potential applications as novel antibiotics (André & Monika 2010). They are well known producer of chitinolytic enzymes and used commercially as a source of these proteins. Additional interest in these enzymes is stimulated by the fact that chitinolytic strains of *Trichoderma* are among the most effective agents of biological control of plant diseases (Harman et al. 1993; Samuels 1996; Spiegel & Chet 1998; Kubicek et al. 2001; Viterbo et al. 2002; Benitez et al. 2004; Navazio et al. 2007; Goswami et al. 2008; Vinale et al. 2009; Karlsson et al. 2010). Chitinases are chitin-degrading enzymes that hydrolyze the β -1, 4-glycosidic bonds between the N-acetyl glucosamine residues of chitin and are widely distributed in nature (Kitamura & Kamei 2003). *Trichoderma* chitinases belong to the glycosyl hydrolase family 18 and can be further grouped into class III and class V. Many chitinase genes from *Trichoderma* have been studied and several laboratories around the world are applying these genes to a variety of bio-control strategies and studying the mechanism of fungal antagonism and mycoparasitism (Markovich & Kononova 2003; Duo-Chuan 2006). For the evaluation of chitinases, insoluble substrates such as tritiated solid chitin, colloidal chitin or chitin covalently coupled to different dyes (chitin red, chitin-azure) can be used. In accordance with the substrate used, the assays are radiometric or photometric. A typical method to evaluate chitinases involves colorimetric quantification (Monreal & Reese 1969) which is time-consuming, less sensitive and not easily applicable to identify poor-chitinolytic microbial strains. Therefore, more simple and rapid assays like colloidal chitin (major carbon source) supplemented in solid basal media resulting in clear halo of chitin-digestion and of the microbial colonies which are measures of the chitinase activity Rojas Avelizapa et al. (2001) are used. However, this method also has a low sensitivity and its results depend on concentration and size of the particles of colloidal chitin, thickness of the media and amount and kind of inoculums. More sensitive techniques require more expensive substrates, which are suitable to study specificity of chitinases more than to select chitinolytic strains (O’Brien & Collwell 1987; McCreath & Gooday 1992; Fra¨ndberg & Schnu¨rer 1994; Barboza Corona et al. 1999). Agrawal and Kotasthane (Agrawal & Kotasthane 2009) proposed a sensitive, easy, reproducible and economic option to determine chitinases (available via Dialog, http://www.isth.info/methods/method.php?method_id=11 ) which was also followed by Kamala and IndiraDevi (Kamala & IndiraDevi 2011; Kamala & IndiraDevi 2012) to evaluate the chitinolytic properties of *Trichoderma* isolates from Manipur (North-East India) against *Pythium aphanidermatum*, *Fusarium oxysporum* and *Rhizoctonia solani*.

Present study describes the screening chitinase activity of *Trichoderma* isolates using two different chitin sources (colloidal chitin derived from *Rhizoctonia* cell wall and commercial chitin) using the simple media supplemented with bromocresol purple (Agrawal & Kotasthane 2009). Screened *Trichoderma* isolates were also assessed spectrophotometrically for N-acetyl-β-D-glucosamine (NAGA) (for total chitinolytic activity) and p-nitrophenol (pNP) (for exochitinase activity) from colloidal chitin supplemented in broth.

## Results and discussion

### Dyeing of basal chitinase detection medium

**Figure 1:**
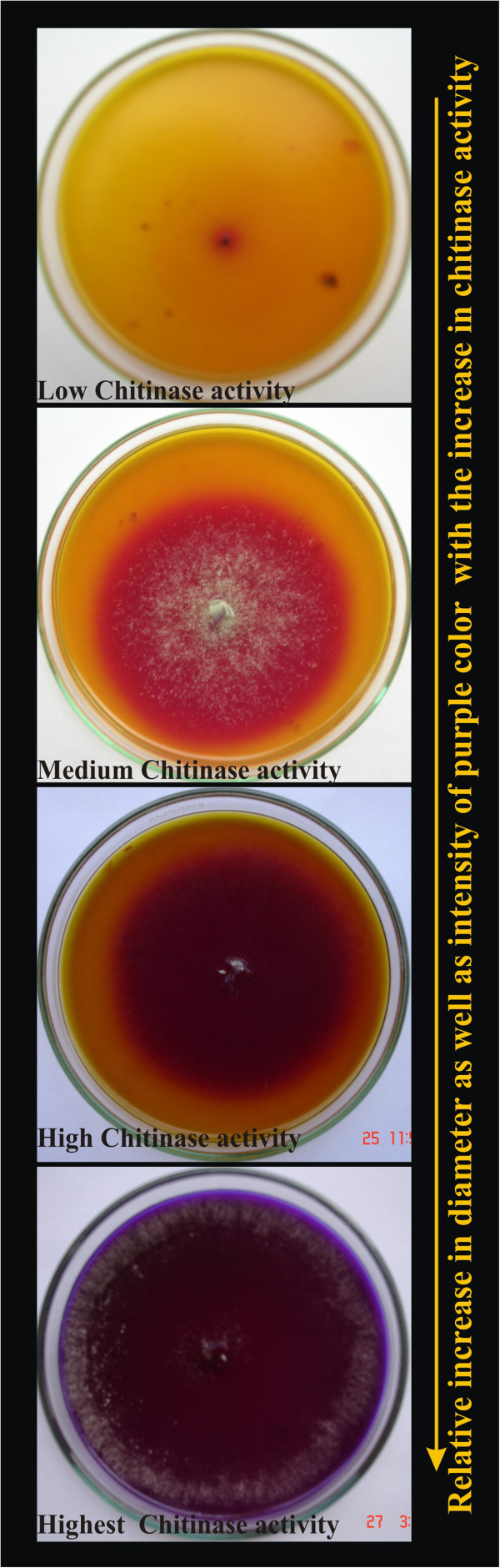
**Screening of*Trichoderma*isolates for chitinase activity on medium supplemented with colloidal chitin.**

Basal chitinase detection medium was directly supplemented with colloidal chitin (4.5g/l) and bromocresol purple (0.15g/l). Resulting substrate had a bright yellow-color, and retained enough bromocresol purple even after pH was adjusted to 4.7 and sterilization at 121°C for 15 min (Figure 1). No complicated protocols for dyeing of the chitinous material and mordant to fix colors were required as per previous reports (Go’mez et al. 2004; (Fen et al. 2006; Wirth & Wolf 1990)).

Separated polysaccharide molecules of hydrochloric acid induced colloidal chitin may therefore, form the basis for hydrogen bonds formation between the chitinous matrix and the dye. Chitobiose, as a minimal repeating unit of chitin is formed by two NAGA molecules linked by a β-1, 4 glycosidic unions and, in every NAGA residue, there are two hydroxyls, one carbonyl and one imine exposed groups that may acts as a reactive binding site for anionic dyes such as bromocresol purple to produce a color-bound complex (Yellow).

### Determination of chitinase activity of *trichoderma* isolates on colloidal chitin supplemented medium

Colloidal chitin media containing bromocresol purple (pH 4.7) when inoculated with chitinolytic *Trichoderma*, resulted in breakdown of chitin into N- acetyl glucosamine causing a corresponding shift in pH towards alkalinity and change of color of pH indicator dye (BCP) from yellow to purple zone surrounding the inoculated fresh culture plugs in the region of chitin utilization (Figure 1). Chitinase activity exhibited by 61 isolates of *Trichoderma* was determined by the diameter of the purple colored zone after 3 days of incubation in the colloidal chitin supplemented agar medium and were classified into different groups (1=no chitinase activity; 2=low chitinase activity; 3=medium chitinase activity and 4=high chitinase activity) (Table 1). Seventeen isolates( # 4, 27, 73, 75, 85, 97, 99, 152, 217, 238, 249, 261, cb4, Gv, Th1, Tv(t) and 231a) gave rapid and highest response in both types of colloidal chitin (derived from *Rhizoctonia* cell wall and commercial chitin) and represented high chitinase activity group(s). Eight (#2, 10, 29, 32, 43, 53, 55, 204) and twelve isolates (#6, 20, 25, 62, 93, 98, 106, 120, 211, 226, 297 and 233b) expressed low and medium chitinase activity respectively. No detectable purple colored zone was observed with isolate #111 on any of the colloidal chitin (derived from *Rhizoctonia* cell wall and commercial chitin) supplemented medium. Variable preference for media supplemented with commercial chitin (Cc) (Himedia) and *Rhizoctonia* cell wall (RCW) derived colloidal chitin was observed among the isolates (Table 1). *Trichoderma* isolates (# 14, 48, 107, 326, 145b1, 40 (Agrawal & Kotasthane 2009)b, 91, 207, 24, 8, 38) showed less preference for chitinase activity on media supplemented with commercial chitin derived colloidal chitin and expressed medium / high chitinase activity on *Rhizoctonia* cell wall derived colloidal chitin whereas isolates # 12, 28, 46, 68, 4, 102, 125b, 114, 173, 202, 243, 332 expressed medium / high chitinase activity on media supplemented with colloidal chitin derived from commercial chitin but expressed low / medium chitinase activity on substrate supplemented with *Rhizoctonia* cell wall derived colloidal chitin (Table 1).

**Table 1:** **Screening of*Trichoderma*isolates for Chitinase production on solid medium supplemented with colloidal chitin derived from*Rhizoctonia*cell wall (RCW) and commercial chitin (Cc)**

***Trichoderma*** isolates showing similar chitinase activity on RCW and Cc derived colloidal chitin				
**Isolate Group**	**NIL**	**Low**	**Medium**	**High**
**A**	-	10, 32	6, 20, 25, 211, 120	Th1, Tv(t), 73, 217, 4
**B**	-	43, 29	-	27
**C**	-	55	62, 98, 297	Gv, 231a , 238, 249
**D**	-	-	93,226	85, 99, 152, 261
**E**	-	2, 53	-	Cb4, 75, 97
**F**	111	204	106, 233b	-
**Isolate Group**	**Low / Medium preference for Cc derived colloidal chitin but Medium / High preference for RCW derived colloidal chitin**			
**A**	14, 207			
**B**	24			
**C**	48, 107, 38			
**D**	145b1, 326			
**E**	-			
**F**	40(1)b, 91, 8			
**Isolate Group**	**Low / Medium preference for RCW derived colloidal chitin but Medium / High preference for Cc derived colloidal chitin**			
**A**	68, 1			
**B**	28			
**C**	12, 202			
**D**	173			
**E**	46, 102			
**F**	114, 243,332, 125b			

A= *T. harzianum / viride*; B= *T. citirinoviride*; C= *T aureoviride*; D= *T. virens*; E= *Isolates belonging to Sec. Longibrachiatum*; F= *Isolates belonging to Sec. Pachybasium.*

Bromocresol Purple (BCP or 5’,5”-dibromo-o-cresolsulfophthalein pKa 6.3), is a halo chromic chemical compound which causes the color of the solution to change depending on the pH and therefore is used to determine pH (acidity or basicity) of the substrate visually. Bromocresol Purple is a detector for hydronium ions (H_3_O^+^) or hydrogen ions (H^+^) in the Arrhenius model. For pH indicators that are weak proteolytes, the Henderson-Hasselbalch equation derived from the acidity constant, states that when pH equals the pKa value of the indicator, both species are present in 1:1 ratio. If pH is above the pKa value, the concentration of the conjugate base is greater than the concentration of the acid, and the color associated with the conjugate base dominates. If pH is below the pKa value, the converse is true.

Usually, the color change is not instantaneous at the pKa value, but there is a pH range where a mixture of colors is present. This pH range falls between the pKa value plus or minus one. If the concentration of the conjugate base (N-acetyl glucosamine+BCP) is ten times greater than the concentration of the acid, their ratio is 10:1, and consequently the pH is pKa+1. Conversely, if there is a tenfold excess of the acid (Colloidal chitin+BCP supplemented acidic media) with respect to the base, the ratio is 1:10 and the pH is pKa–1. Chitin agar plate has been used earlier for isolating chitinolytic microorganisms and observing clear zone around the colony of microorganisms ((Wirth & Wolf 1990; Cody 1989); Rojas Avelizapa et al. 2001). Previous attempt to synthesize a remazol brilliant blue (RBB) derivative of chitosan resulted in a relatively poorly soluble polymer (Fen et al. 2006) which rendered difficult its application to direct screening of microbial colonies on agar media but soluble RBB-chitosan (lower molecular weight derivative) was particularly suitable for the detection of chitosanase positive organisms on agar media (Brzezinski et al. 2010). However, these methods have low sensitivity and its results depend on concentration and size of the particles of colloidal chitin, thickness of the media and the amount and kinds of inoculum. Glycol chitin and indicators such as Calcoflour White M2R, Flourescein isothiocyanate, Rhodamine B etc. to screen hyperchitinase producing bacteria and fungus has been reported by (Vaidya et al. 2003). More sensitive techniques require more expensive substrates, which are suitable to study specificity of chitinases more than to select chitinolytic strains (O’Brien & Collwell 1987; McCreath & Gooday 1992; Fra¨ndberg & Schnu¨rer 1994; Barboza Corona et al. 1999). The evaluation of chitinases using pH indicator dye bromocresol purple as proposed by Agrawal and Kotasthane (Agrawal & Kotasthane 2009) (available via Dialog, http://www.isth.info/methods/method.php?method_id=11. Posted on 2009-08-04 at ISTH) has the following advantages: (A) the soluble substrate with pH indicator dye (BCP) for the assay of chitinase activity on solid media is sensitive, easy, reproducible semi-quantitative enzyme diffusion plate assay and economic option to determine chitinases. (B) its preparation is easy and inexpensive; (C) the possibility of interference of BCP is minimal under the environmental conditions used ordinarily in the chitinase assays; (E) as colloidal chitin (CC)-BCP is not toxic for microorganisms, it can be added simultaneously as a carbon source and as a chitinase inducer in the culture media. This fact allows a simpler, fast and accurate one-step process for the selection of chitinolytic microorganisms and was therefore also followed by Kamala and IndiraDevi (Kamala & IndiraDevi 2011; Kamala & IndiraDevi 2012) to evaluate the chitinolytic properties of *Trichoderma* isolates from Manipur (North-East India) against *Pythium aphanidermatum*, *Fusarium oxysporum* and *Rhizoctonia solani*.

### Spectrophotometric determination of chitinase activity of *Trichoderma* isolates

#### Total chitinolytic activity

**Figure 2:**
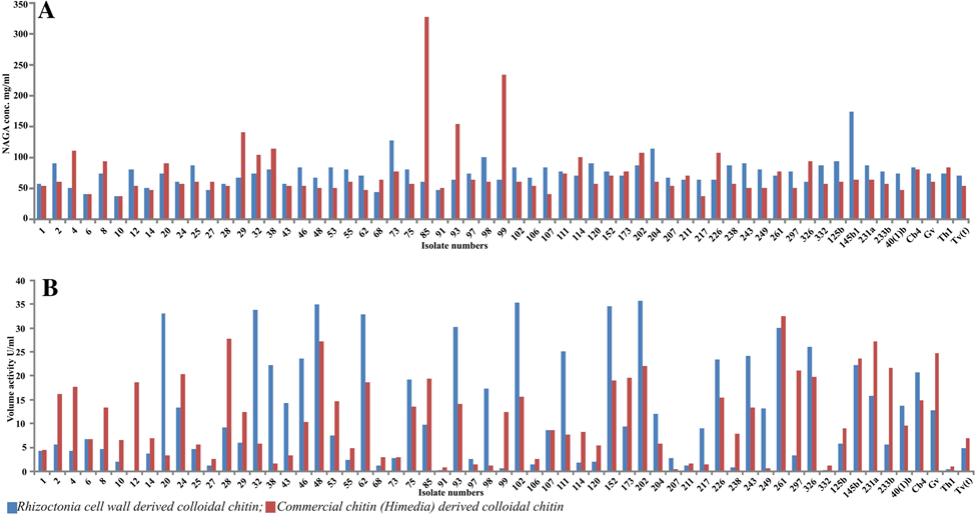
**A. Chitinolytic activity of culture filtrates of*Trichoderma*isolates grown on broth medium supplemented with*Rhizoctonia*cell wall and Commercial chitin derived colloidal chitin. B.** Exochitinase activity of culture filtrates of *Trichoderma* isolates grown on broth medium supplemented with *Rhizoctonia* cell wall and Commercial chitin derived colloidal chitin.

Total chitinolytic activity was assayed by measuring the release of reducing saccharides from colloidal chitin. Standard curve generated with N-acetyl-β-D-glucosamine (NAGA) was used to determine reducing saccharide concentration. Chitinolytic activity was expressed in terms of the concentration of NAGA (mg/ml) released in colloidal chitin (derived from *Rhizoctonia* cell wall and commercial chitin) supplemented media. Released NAGA concentration ranged from 37.67 (isolate #10) to 174.33 (isolate #145b1) mg/ml and 37.67(isolate #10) to 327.67(isolate #85) mg/ml in *Rhizoctonia* cell wall and commercial chitin derived colloidal chitins respectively (Table 2; Figure 2A).

**Table 2:** **Spectrophotometric determination of Chitinolytic and Exochitinase activity of*Trichoderma*isolates in media supplemented with colloidal chitin derived from*Rhizoctonia*cell wall (RCW) and Commercial chitin (Cc)**

Isolate No.	NAGA conc. (mg/ml)		Volume activity (U/ml X 10^-3^)	
	RCW	Cc	RCW	Cc
**GROUP A*(T. harzianum / viride)***				
1	57.67	54.33	4.26	4.54
4	51	111	4.26	17.89
6	41	41	6.82	6.76
10	37.67	37.67	1.99	6.65
14	51	47.67	3.69	7.04
20	74.33	91	33.17	3.41
25	87.67	61	4.66	5.62
32	74.33	104.33	34.02	5.85
68	44.33	64.33	1.25	2.95
73	127.67	77.67	2.84	3.12
120	91	57.67	2.10	5.57
207	67.67	54.33	2.89	0.63
211	64.33	71	1.31	1.76
217	64.33	37.67	9.09	1.48
Th1	74.33	84.33	0.63	1.19
Tv(t)	71	54.33	4.89	7.1
Mean	67.46	65.58	7.37	5.09
	(5.59)	(5.66)	(2.62)	(1.02)
P	0.4837^ns^		0.0004^s^	
**Group B (*T. citirinoviride*)**				
24	61	57.67	13.52	20.51
27	47.67	61	1.36	2.73
28	57.67	54.33	9.26	27.83
29	67.67	141	5.96	12.49
43	57.67	54.33	14.37	3.41
Mean	58.34	73.67	8.89	13.39
	(3.23)	(16.88)	(2.42)	(4.86)
P	0.0037^s^		0.102^ns^	
**Group C (*T. aureoviride*)**				
12	81	54.33	0.17	18.80
38	81	114.33	22.44	1.76
48	67.67	51	35.16	27.21
55	81	61	2.49	4.88
62	71	47.67	33.06	18.69
98	101	61	17.44	1.25
107	84.33	41	8.69	8.80
202	87.67	107.67	35.78	22.15
249	81	51	13.18	0.85
297	77.67	51	3.35	21.24
231a	87.67	64.33	15.90	27.38
Gv	74.33	61	12.89	24.77
Mean	81.28	63.78	16.71	14.82
	(2.51)	(6.66)	(3.64)	(3.04)
P	0.0015^s^		0.2799^ns^	
**Group D (*T. virens*)**				
85	61	327.67	9.88	19.54
93	64.33	154.33	30.33	14.26
99	64.33	234.33	0.68	12.55
152	77.67	71	34.76	19.09
173	71	77.67	9.43	19.77
226	64.33	107.67	23.57	15.62
261	71	77.67	30.16	32.60
326	61	94.33	26.18	19.82
145b1	174.33	64.33	22.38	23.69
Mean	78.78	134.33	20.82	19.66
P	0.0091^s^		0.0282^s^	
**Group E (*Isolates of Sec.L ongibrachiatum*)**				
Cb4	84.33	81	20.85	14.88
2	91	61	5.62	16.36
46	84.33	54.33	23.69	10.51
53	84.33	51	7.61	14.77
75	81	57.67	19.31	13.69
97	74.33	64.33	2.73	1.48
102	84.33	61	35.5	15.68
Mean	83.38	61.48	16.47	12.48
	(1.89)	(3.67)	(4.44)	(1.96)
P	0.065^ns^		0.0342^s^	
**Group F (*Isolates of Sec. Pachybasium*)**				
8	74.33	94.33	4.71	13.41
91	47.67	51	0.45	0.91
106	67.67	54.33	1.54	2.61
111	77.67	74.33	25.28	7.78
114	71	101	1.82	8.41
204	114.33	61	12.16	5.79
238	87.67	57.67	1.02	7.89
243	91	51	24.31	13.41
332	87.67	57.67	0.34	1.42
125b	94.33	61	5.79	9.09
233b	77.67	57.67	5.62	21.87
40(1)b	74.33	47.67	13.92	9.71
Mean	80.45	64.06	8.08	8.53
	(4.75)	(4.95)	(2.56)	(1.69)
P	0.4463^ns^		0.0886^ns^	

*Figures in parenthesis represent standard error.*

^*s*^*=significant(P-value<0.05);*^*ns*^*= non-significant (P-value>0.05).*

Amount of released NAGA (mg/ml) in colloidal chitin supplemented media by individual isolates of *Trichoderma* spp. formed the basis to categorize them in 1) Low (NAGA conc. 30–60 mg/ml) 2) Medium (NAGA conc. 61–80 mg/ml) and 3) High (NAGA conc. 81 mg/ml and above) chitinase producers. Three isolates (#38, Cb4 and 202) were identified as high chitinase producers where as seven isolates belonged to each low (#1, 6, 10, 14, 28, 43 and 91) and medium (#97, 111, 152, 173, 211, 261, and Gv) chitinase activity groups. The isolates expressed differential chitinase activity on media supplemented with *Rhizoctonia* cell wall and commercial chitin derived colloidal chitins. Commercial chitin derived colloidal chitin supplemented broth was less preferred chitin source (by only 14 isolates) as compared to *Rhizoctonia* cell wall derived chitin which was more potent in inducing total chitinolytic response in 30 isolates *Trichoderma* spp. Fourteen *Trichoderma* isolates expressed higher preference and an increased release of NAGA (medium / high chitinase) on media supplemented with colloidal chitin derived from commercial chitin than *Rhizoctonia* cell wall derived colloidal chitin (Table 3).

**Table 3:** **Chitinolytic activity of culture filtrates of*Trichoderma*isolates grown on media (broth) supplemented with colloidal chitin derived from*Rhizoctonia*cell wall (RCW) and Commercial chitin (Cc)**

***Trichoderma*** isolates showing similar Chitinolytic activity (NAGA conc.)			
**Isolate Group**	**Low**	**Medium**	**High**
**A**	1, 6, 10, 14	211	-
**B**	28,43	-	-
**C**	-	Gv	38, 202
**D**	-	152, 261 173	-
**E**	-	97	Cb4
**F**	91	111	-
**Isolate Group**	**Low / Medium preference for Cc derived colloidal chitin but Medium / High preference for RCW derived colloidal chitin**		
**A**	Tv(t), 120, 217, 207, 25, 73		
**B**	24		
**C**	12, 62, 48, 107, 249, 297,55, 98, 231a		
**D**	145b1		
**E**	46, 53, 75, 2, 102		
**F**	40(1)b, 106, 233b, 238, 243, 332, 125b, 204		
**Isolate Group**	**Low / Medium preference for RCW derived colloidal chitin but Medium / High preference for Cc derived colloidal chitin**		
**A**	4, 68, 20, 32, Th1		
**B**	27, 29		
**C**	-		
**D**	85, 93, 99, 226, 326		
**E**	-		
**F**	8, 114		

*Chitinase activity (NAGA conc.) Low=(30–60 mg/ml); Medium=(61–80 mg/ml); High=(81 mg/ml and above); A= T. harzianum / viride; B= T. citirinoviride; C= T aureoviride; D= T. virens; E= Isolates belonging to Sec. Longibrachiatum; F= Isolates belonging to Sec. Pachybasium.*

#### Exochitinase activity

Similarly, exochitinase activity (N-acetyl-β-D-glucosaminidase) was measured as release of p-nitrophenol (pNP) from p-nitrophenyl-N-acetyl-β-D-glucosaminide (pNPg) The volume activity of pNP ranged from 0.17 to 35.78 X 10^-3^ U/ml and 0.62 to 32.6 X 10^-3^ U/ml in *Rhizoctonia* cell wall and commercial chitin derived colloidal chitins respectively. Minimum volume activity of pNP was observed to be 0.17 X 10^-3^ U/ml for the isolate #12 and 0.62 X 10^-3^ U/ml for the isolate #207 respectively in *Rhizoctonia* cell wall and commercial chitin derived colloidal chitins whereas maximum was for isolates #202 and #261 respectively (Table 2; Figure 2B).

Volume activity of pNP formed the basis to establish exochitinase activity in the reaction mixture and the differential responses exhibited by different isolates were broadly categorized as 1) Low (Volume activity U/ml 0.1 to 7.0 X 10^-3^) 2) Medium (Volume activity U/ml 7.1 to 17 X 10^-3^) and 3) High (Volume activity U/ml 17 X 10^-3^ and above). Seven isolates (# 62, 202, 48, 152, 326, 145b1, 261) were identified as high chitinase producers where as sixteen (# 1, 6, 10, 25, 27, 55, 68, 73, 91, 97, 106, 120, 207, 211, 332, Th1 ) and three (#107, 53, 40 (Agrawal & Kotasthane 2009)b) isolates belonged to low and medium chitinase activity groups. The isolates expressed differential chitinase activity on media supplemented with *Rhizoctonia* cell wall and commercial chitin derived colloidal chitins Sixteen *Trichoderma* isolates showed higher preference and expressed medium / high chitinase activity on *Rhizoctonia* cell wall derived colloidal chitin whereas *Trichoderma* 19 isolates expressed higher preference and an increased volume activity (medium / high chitinase) on media supplemented with colloidal chitin derived from commercial chitin (Table 4).

**Table 4:** **Exochitinase (N-acetyl-β-D-glucosaminidase) activity of culture filtrates of*Trichoderma*isolates grown on media (broth) supplemented with colloidal chitin derived from*Rhizoctonia*cell wall (RCW) and Commercial chitin (Cc)**

***Trichoderma*** isolates showing similar Exochitinase activity (Volume activity)			
**Group**	**Low**	**Medium**	**High**
**A**	207, Th1, 211, 68, 73, 1, 120, 25, 10, 6	-	-
**B**	27	-	-
**C**	55	107	62, 202, 48
**D**	-	-	152, 326, 145b1, 261
**E**	97	53	-
**F**	91, 332, 106	40(1)b	-
**Isolate Group**	**Low / Medium preference for Cc derived colloidal chitin but Medium / High preference for RCW derived colloidal chitin**		
**A**	217, 20, 32		
**B**	43		
**C**	249, 98, 38		
**D**	93, 226		
**E**	46, 75, 102, Cb4		
**F**	204, 111, 243		
**Isolate Group**	**Low / Medium preference for RCW derived colloidal chitin but Medium / High preference for Cc derived colloidal chitin**		
**A**	14, 4, Tv(t)		
**B**	29, 28, 24		
**C**	12, 297, GV, 231a		
**D**	99, 173, 85		
**E**	2		
**F**	238, 114, 8, 233b, 125b		

*Volume activity Low =(0.1 to 7.0 X 10*^*-3*^*U/ml); Medium=(7.1 to 17 X 10*^*-3*^*U/ml); High =(above 17 X 10*^*-3*^*U/ml); A= T. harzianum / viride; B= T. citirinoviride; C= T aureoviride; D= T. virens; E= Isolates belonging to Sec. Longibrachiatum; F= Isolates belonging to Sec. Pachybasium.*

Three isolates of *Trichoderma* spp. (#38, Cb4 and 202) were identified as high for total chitinolytic activity and seven isolates (# 62, 202, 48, 152, 326, 145b1, 261) were identified as high exochitinase (N-acetyl-β-D-glucosaminidase) producers following spectrophotometric determination. Isolates # Cb4, 152, 261 were also identified as high chitinase producers on solid medium. Chitinase activity of *Trichoderma* isolates as determined in plates and by spectrophotometric assay is not always in agreement with the information obtained in a secondary screening/ spectrophotometric assays. It was observed that the isolates with poor or low chitinase activity in solid medium showed medium or high chitinolytic and / or volume activity spectrophotometrically. This disagreement in preliminary data and secondary screening might be due to the quantitative evaluations of the enzyme secreted by every strain in a liquid medium, along different times of incubation. In addition, assays using different dilutions of the samples are required when the level of the enzyme is high, or extending the time of incubation if the amount of enzyme is low. The results of total chitinolytic activity assayed by measuring the release of N-acetyl-β-D-glucosamine (reducing saccharides) from colloidal chitin and N-acetyl-β-D-glucosaminidase (exochitinase) activity measured as release of p-nitrophenol (pNP) from p-nitrophenyl-N-acetyl-β-D-glucosaminide (pNPg) are in agreement with those of (El-Katatny et al. 2000), Tweddell et al. (Tweddell et al. 1994) and Calistru et al. (1997) who reported the use of crude culture filtrates of *T. harzianum* possessing β-1,3-glucanase and chitinase activities. It revealed the ability to release reducing sugars (glucose, GlcNAc) from dried or fresh mycelium of the phytopathogenic fungus *S. rolfsii*. Harman et al. (1993) measured and monitored chitinase activity spectrophotometrically as the release of the p-nitrophenol (pNP) from p-nitrophenyl-N-acetyl-D-glucosaminide. The observations of the present investigation are supported by those reported by De la Cruz et al. (1992) and Lorito et al. (1994a) that the production of the hydrolytic enzymes has been affected by culture conditions and by the host. The production of extracellular chitinases and ß-glucanases was produced in the presence of phytopathogen cell walls as carbon source, suggesting that these substrate can also act as inducers of the synthesis of the lytic enzymes.

## Conclusions

The results of this study clearly show that large inter strain and inter species differences exist in both total chitinolytic and exochitinase activity of *Trichoderma* isolates. However, there appears to be no common pattern of chitinase activity in the media supplemented with *Rhizoctonia* cell wall and commercial chitin derived colloidal chitin. Some isolates, e.g. *T. harzianum/viride* (#20 & 32) and *T. aureoviride* (#38, 98 & 249) showed highest exochitinase activity whereas *T. aureoviride* (#12, 107 & 249); isolates of Sec. *Longibrachiatum* (#46, 53 & 75) and isolates of Sec. *Pachybasium* (# 238, 243 & 332) produced highest total chitinolytic activity in medium supplemented with colloidal chitin derived from *Rhizoctonia* cell wall, while others showed little difference in chitinase activity irrespective of colloidal chitin type. There appears to be no direct relationship between levels of NAGA and pNP produced by individual *Trichoderma* isolates. For example, *T. harzianum/viride* isolate #73 produced one of the highest total chitinase activities (NAGA conc.) recorded but one of the lowest exochitinase activities in medium supplemented with RCW derived colloidal chitin.

In general, *Rhizoctonia* cell wall derived colloidal chitin was able to induce chitinolytic activity in more number of isolates (38 isolates of *Trichoderma* spp.) and belonged to medium / high chitinolytic category where as the reaction mixture containing commercial chitin derived colloidal chitin induced total chitinolytic as well as exochitinase activity in 26 isolates of *Trichoderma* spp. (Table 5). This suggests that there has been a shift in the balance of the organism’s metabolic processes towards production of lytic enzymes under these conditions. Sivan and Chet (1989) reported that the production of extracellular β-(André & Monika 2010; Agrawal & Kotasthane 2009)-glucanases, chitinases and proteinase increases significantly when *Trichoderma* spp. are grown in media supplemented with either autoclaved mycelium or isolated purified host fungal cell walls. Several lines of similar evidences indicate the use of different substrates such as p-nitro phenyl keto oligomers, differentially purified chitins or fungal cell walls, 4-methyl umbelliferil derivatives, blue substrate etc. to assay, purify and characterize chitinolytic enzymes from *Trichoderma* (Harman et al. 1993; Lorito et al. 1994a; Ulhoa & Peberdy 1993; De la Cruz et al. 1993; Schickler et al. 1998). Nutrient content of medium is one of the determinant of the levels of lytic enzymes produced and the presence of cell wall material has a significant influence on the production of chitinase by many *Trichoderma* isolates.

**Table 5:** **Preference for chitinase activity (NAGA conc.) on media supplemented with*Rhizoctonia*cell wall (RCW) and Commercial chitin (Cc) derived colloidal chitin**

Isolate Group	Low / Medium preference for Cc derived colloidal chitin but Medium / High preference for RCW derived colloidal chitin	Low / Medium preference for RCW derived colloidal chitin but Medium / High preference for Cc derived colloidal chitin
**A**	20, 32, 25, 73, 120, 207, 217, Tv(t)	4, 14, 20, 32, 68, Th1, Tv(t)
**B**	24, 43	24, 27, 28, 29
**C**	12, 38, 48, 55, 62, 98, 107, 249 297, 231a	12, 231a, 297, Gv
**D**	93, 145b1, 226	85, 93, 99, 173, 226, 326
**E**	2, 46, 53, 75, 102, Cb4	2
**F**	40(1)b, 106, 111, 125b, 204, 233b, 238, 243, 332	8, 114, 125b, 233b, 238
**Total**	38	26

*A= T. harzianum / viride; B= T. citirinoviride; C= T aureoviride; D= T. virens; E= Isolates belonging to Sec. Longibrachiatum; F= Isolates belonging to Sec. Pachybasium.*

## Materials and methods

### Fungal isolates

Sixty one *Trichoderma* isolates from rhizosphere and non rhizosphere soil samples collected from different geographical locations of Chhattisgarh were characterized (as per key identification parameters Gams and Bissets, (Gams & Bissett 1998)) and maintained in potato dextrose agar (PDA, Himedia) slants in the Department of Plant Molecular Biology & Biotechnology, IGKV, Raipur, India. Of 61 *Trichoderma* isolates, 16, 5, 12, 9, 7 and 12 isolates belonged to species groups **A** (*T. harzianum / T. viride)*, **B** (*T. citrinoviride)*, **C** (*T. aureoviride)*, D (*T. virens)*, **E** (Sec. *Longibrachiatum*) and **F** (Sec. *Pachybasium*) respectively. *Rhizoctonia* sp. used for colloidal chitin preparation was isolated from the sick soil of the rice field.

### Preparation of colloidal chitin

Colloidal chitin was prepared from *Rhizoctonia* cell wall and commercial chitin (Himedia) by the method of Roberts and Selitrennikoff (Roberts & Selitrennikoff 1988) with a few modifications and supplemented in the chitinase assay medium as a sole carbon source. Acid hydrolysis of chitin was done in conc. HCl by constant stirring using a magnetic stirrer at 4°C (refrigerator) overnight, which was followed by extraction of colloidal chitin in 2000 ml of ice-cold 95% ethanol neutralization kept at 26°C for overnight. It was then centrifuge at 3000 rpm for 20 min at 4°C. Pellet was washed with sterile distilled water by centrifugation at 3000 rpm for 5 min at 4°C till the smell of alcohol was completely removed. The colloidal chitin obtained had a soft, pasty consistency with 90–95% moisture and was stored at 4°C until further use.

### Agar medium for detection of chitinase-positive microorganisms

Chitinase detection medium consisted of a basal medium comprising (per liter) 0.3 g of MgSO4.7H2O, 3.0 g of (NH_4_)_2_SO_4_, 2.0 g of KH_2_PO_4_, 1.0 g of citric acid monohydrate, 15 g of agar, 200 μl of Tween-80, 4.5 g of colloidal chitin and 0.15 g of bromocresol purple; pH was adjusted to 4.7 and then autoclaved at 121°C for 15 min. Lukewarm medium was poured in petri-plates and allowed to solidify. Fresh culture plugs of the isolates to be tested for chitinase activity were inoculated into the medium and incubated at 25±2°C and were observed for colored zone formation.

### Spectrophotometric determination of chitinase activity of *Trichoderma* isolates

Culture plugs containing young actively growing mycelium of *Trichoderma* isolates were inoculated in colloidal chitin (derived from *Rhizoctonia* cell wall chitin and commercial chitin) supplemented broth (without bromocresol purple) and incubated at 28°C for 5 days at 200 rpm. Cultural filtrates obtained by filtering through Whatman No. 1 filter paper were stored at −20°C until further use. Filtrates were analyzed through spectrophotometric assay was performed for total chitinolytic and N-acetyl-β-D-glucosaminidase activities.

#### Total chitinolytic activity

Total chitinolytic activity was assayed by measuring the release of reducing saccharides from colloidal chitin. A reaction mixture containing 1 ml of culture supernatant, 0.3 ml of 1 M sodium acetate buffer (SA-buffer), pH 4.6 and 0.2 ml of colloidal chitin was incubated at 40°C for 20 h and then centrifuged at 13,000 rpm for 5 min at 6°C. After centrifugation, an aliquot of 0.75 ml of the supernatant, 0.25 ml of 1% solution of dinitrosalycilic acid in 0.7 M NaOH and 0.1 ml of 10 M NaOH were mixed in 1.5 ml micro centrifuge tubes and heated at 100°C for 5 min. Absorbance of the reaction mixture at *A*_582_ was measured after cooling to room temperature (Miller 1959) Calibration curve with N-acetyl-β--D-glucosamine (NAGA) was used as a standard to determine reducing saccharide concentration. Under the assay conditions described, a linear correlation between *A*_582_ and NAGA concentration was found in the interval of 40–800 mg/ml NAGA. Chitinolytic activity was estimated in terms of the concentration (mg/ml) of NAGA released.

#### Exochitinase activity

N-acetyl-β-D-glucosaminidase (exochitinase) activity was measured and monitored spectrophotometrically as the release of p-nitrophenol (pNP) from p-nitrophenyl-N-acetyl-β-D-glucosaminide (pNPg) A mixture of 25 μl of culture filtrate, 0.2 ml of pNPg solution (1 mg pNPg ml^-1^), and 1 ml of 0.1 M SA-buffer (pH 4.6) was incubated at 40C for 20h and then centrifuged at 13,000 rpm. An aliquot of 0.3 ml of 0.125 M Sodium tetraborate–NaOH buffer (pH 10.7) was added to 0.6 ml of supernatant, absorbance at 400 nm (*A*_400_) was measured immediately after mixing and pNP concentration (in terms of Volume Activity) in the solution was calculated using the pNP molar extinction coefficient (18.5 mM^-1^ - cm^-1^) with the help of following formula:

where,

V_t_ = Total volume (900 μl); Vs = Sample volume (25 μl); 18.5 = Millimolar extinction coefficient of p-nitrophenol under the assay condition (cm^2^/micromole); 1.0 = Light path length (cm); t = Reaction time (20 hours = 1200 minutes); df = Dilution factor (Miller 1959).
